# Novel DNA Repair Inhibitors Targeting XPG to Enhance Cisplatin Therapy in Non-Small Cell Lung Cancer: Insights from In Silico and Cell-Based Studies

**DOI:** 10.3390/cancers16183174

**Published:** 2024-09-16

**Authors:** Rita Manguinhas, Patrícia A. Serra, Nuno Gil, Rafael Rosell, Nuno G. Oliveira, Rita C. Guedes

**Affiliations:** 1 Research Institute for Medicines (iMed.ULisboa), Faculty of Pharmacy, Universidade de Lisboa, 1649-003 Lisboa, Portugal; rmanguinhas@edu.ulisboa.pt (R.M.); pserra@egasmoniz.edu.pt (P.A.S.); 2 Lung Unit, Champalimaud Clinical Centre (CCC), Champalimaud Foundation, 1400-038 Lisboa, Portugal; nuno.gil@fundacaochampalimaud.pt; 3 Egas Moniz Interdisciplinary Research Center, Instituto Universitário Egas Moniz, 2829-511 Caparica, Portugal; 4 Dr. Rosell Oncology Institute, 08028 Barcelona, Spain; rrosell@oncorosell.com; 5 Institute Germans Trias i Pujol, 08916 Badalona, Spain

**Keywords:** DNA repair inhibitors, XPG protein, cisplatin, NSCLC, virtual screening

## Abstract

**Simple Summary:**

Non-small cell lung cancer (NSCLC) is marked by low survival and resistance to platinum-based chemotherapy. Recent studies have emphasized the critical role of DNA repair mechanisms in NSCLC tumorigenesis and response to treatment. XPG endonuclease, a crucial component of the Nucleotide Excision Repair (NER) pathway, has emerged as a promising biomarker of clinical outcome in advanced NSCLC and its downregulation improved cisplatin efficacy by increasing DNA damage. However, so far, no study has been carried out with the purpose of identifying XPG inhibitors. This work thus aims to discover potential small-molecule inhibitors of XPG to be used in combination with cisplatin therapy to enhance its efficacy in NSCLC patients.

**Abstract:**

NSCLC is marked by low survival and resistance to platinum-based chemotherapy. The XPG endonuclease has emerged as a promising biomarker for predicting the prognosis of cisplatin-treated patients and its downregulation having been reported to increase cisplatin efficacy. This study presents an integrated strategy for identifying small molecule inhibitors of XPG to improve cisplatin therapy in NSCLC. A structure-based virtual screening approach was adopted, including a structural and physicochemical analysis of the protein, and a library of small molecules with reported inhibitory activities was retrieved. This analysis identified Lys84 as a crucial residue for XPG activity by targeting its interaction with DNA. After molecular docking and virtual screening calculations, 61 small molecules were selected as potential XPG inhibitors, acquired from the ChemBridge database and then validated in H1299 cells, a NSCLC cell line exhibiting the highest *ERCC5* expression. The MTS assay was performed as a first screening approach to determine whether these potential inhibitors could enhance cisplatin-induced cytotoxicity. Overall, among the eight compounds identified as the most promising, three of them revealed to significantly increase the impact of cisplatin. The inherent cytotoxicity of these compounds was further investigated in a non-tumoral lung cell line (BEAS-2B cells), which resulted in the identification of two non-cytotoxic candidates to be used in combination with cisplatin in order to improve its efficacy in NSCLC therapy.

## 1. Introduction

DNA repair encloses a plethora of pathways that are triggered to maintain genetic stability and integrity when cells are exposed to DNA-damaging agents. These complementary repair pathways are also crucial for cells to manage the endogenous DNA lesions that are continuously formed [1,2,3]. Deregulation of these pathways can lead to the initiation and progression of cancer. Overexpression of several key players in DNA repair mechanisms has been identified as a biomarker of cancer progression and is associated with poorer prognosis [4]. Alterations in these pathways can contribute to either hypersensitivity or resistance of cancer cells to genotoxic agents. Since many current therapies depend on inducing cell death through direct or indirect DNA damage (e.g., ionizing radiation and chemotherapeutic agents), targeting specific components of DNA repair pathways can increase tumor sensitivity for these standard cancer treatments [5,6,7,8]. This approach has led to an emerging field in cancer research that focuses on using pharmacological inhibitors of DNA repair enzymes/pathways to boost chemo/radiotherapy or as monotherapy [9,10,11,12,13].

Among the several pathways involved in DNA repair, the nucleotide excision repair (NER) pathway is responsible for removing bulky, helix-distorting DNA lesions, such as those caused by UV light, environmental mutagens (e.g., benzo[a]pyrene or aromatic amines), and chemotherapeutic agents (e.g., platinum-based drugs, mitomycin C, carmustine, and nitrogen mustards) [14]. The NER pathway repairs, among others, pyrimidine dimers, bulky DNA adducts, DNA–DNA crosslinks, and replication lesions [7]. Deficiencies in NER components result in xeroderma pigmentosum (XP), a skin cancer-prone inherited disorder characterized by increased UV sensitivity and mutagenicity in cells [15]. Recently, the absence of functional excision repair cross-complementation group 1-xeroderma pigmentosum complementation group F (ERCC1-XPF) or xeroderma pigmentosum complementation Group G (XPG) was reported to lead to a stable and prolonged binding of the transcription/DNA repair factor TFIIH to DNA damage, this being correlated with senescence in human cells [16].

While NER is pivotal in maintaining global stability, the pharmacological modulation of this pathway holds significant clinical relevance in therapeutic contexts where DNA repair capacity may determine the treatment outcomes. Within the cancer research field of “DNA repair targeted therapy”, several promising drugs have emerged, particularly targeting poly [ADP-ribose] polymerase 1 (PARP1), and to a lesser extent O^6^-methyltransferase, ataxia telangiectasia mutated (ATM)/ DNA-dependent protein kinase (DNA-PK), and human apurinic/apyrimidinic endonuclease 1 (APE1) [5,7,17,18]. However, there is a clear need for the development of improved NER inhibitors to be used in cancer [19]. Computer-Aided Drug Design (CADD) approaches have also been utilized, primarily targeting the ERCC1-XPF complex, the xeroderma pigmentosum complementation group A (XPA), and the replication protein A (RPA), resulting in the identification of some direct inhibitors [18,19,20,21].

As critical components of the NER pathway, the structure-specific endonuclease activities of the ERCC1-XPF complex and the XPG protein are essential for repairing various DNA lesions, including intra-strand and inter-strand crosslinks (ICLs). The ERCC1-XPF complex functions as a heterodimer, where XPF has the nuclease function of incising the DNA 5′ to the lesion. ERCC1, though catalytically inactive, regulates DNA−protein and protein−protein interactions. Given its involvement in repairing DNA damage induced by chemotherapeutics, the ERCC1-XPF complex is a promising druggable target to enhance chemotherapy efficacy and overcome resistance [22,23]. Overexpression of ERCC1-XPF has been linked with poorer prognosis and reduced response to chemotherapeutics in various cancers, including ovarian [24,25], testicular [26], non-small cell lung cancer (NSCLC) [27,28,29], and squamous cell carcinoma [30]. Altogether, the relevance of this target justifies the interest of the scientific community. Despite numerous attempts to develop novel ERCC1-XPF inhibitors, many candidates have exhibited high off-target cytotoxicity, poor pharmacokinetic and physicochemical profiles, and limited potency [31,32,33,34,35,36,37,38]. Our most recent study also identified several promising ERCC1-XPF inhibitors, contributing to the ongoing efforts in this field [39].

The importance of the NER pathway extends beyond ERCC1-XPF, as other targets within this pathway are equally critical. In this context, XPG is the endonuclease responsible for making the 3′ incision at sites of DNA damage. Knockdown studies of XPF and XPG (encoded by the *ERCC5* gene) have shown a fivefold decrease in the repair efficiency of cisplatin/oxaliplatin-induced DNA damage, suggesting a potential for combinatory therapy [40]. This rationale relies on the fact that these endonucleases function sequentially and are indispensable for the completion of NER. Moreover, *ERCC5* downregulation has been identified as a novel prognostic biomarker for platinum-treated ovarian cancer patients [41]. However, so far, no study has been carried out with the purpose of identifying XPG inhibitors.

Lung cancer (LC) is the leading cause of cancer-related deaths for both men and women, with NSCLC accounting for approximately 85% of all LC cases. Unlike the steady increase in survival rates observed for most cancers, progress has been limited for LC, with the current 5-year relative survival rate hovering around 20% [42,43]. Platinum-based adjuvant therapies are typically the first-line treatment, yet resistance to these therapies is common [44]. Given the results observed in other types of cancers and the absence of existing inhibitors, this study aims to identify XPG inhibitors to use in combination therapy with cisplatin in NSCLC patients to improve the clinical outcomes. This study involved characterizing the biological target’s structure, compiling reported inhibitors, selecting an appropriate docking protocol, and conducting a virtual screening campaign. Post-screening, potential inhibitors were acquired from the ChemBridge database and tested in an NSCLC cell line with high *ERCC5* expression. A pre-clinical safety assessment was also performed using a non-tumoral lung cell line to identify the safest inhibitors capable of enhancing cisplatin-induced cytotoxicity.

Our findings illustrate the significance of focusing on key small molecule/protein features to guide the discovery of XPG function modulators. This research provides valuable insights that could lead to the identification of potential inhibitors for use in combination with cisplatin in treating NSCLC.

## 2. Materials and Methods

### 2.1. Computational Studies

#### 2.1.1. Protein Structure Selection and Preparation

Protein Data Bank (PDB) [45] had seven 3D structures for the XPG protein listed when this study first began. Of these, two identified by PDB IDs, 5EKG and 5EKF [46], were obtained from Mus musculus, and the other five represented the human variant of XPG. Interestingly, two of the structures involved interactions with DNA, but none of these structures contained small-molecule inhibitors. Five structures featuring the XPG endonuclease catalytic domain were selected for simulations and analysis, specifically PDB IDs 6VBH [47], 6TUR, 6TUS, 6TUX, and 6TUW [48]. These structures were retrieved from PDB and prepared using the Molecular Operating Environment (MOE v.2020.0901, MOE from now on) [49] structure preparation module used in previous works by our group [50]. After adding hydrogen atoms, the Protonate-3D tool in the MOE software [49] was used to assign the proper protonation states at pH 7.4. Water molecules and non-essential molecules were eliminated.

#### 2.1.2. Ligand Structure Selection and Preparation

A comprehensive search of the ChEMBL28 database [51] was conducted to retrieve reported small molecules that could target XPG. As of April 2022, 64 entries were identified. These molecules’ SMILES notation was first converted into 3D structures. At pH 7.4 and 300 K, each molecule was prepared with the MOE’s Protonate 3D tool. To guarantee an “ideal initial” conformation, the molecules went through energy minimization utilizing the PM7 semi-empirical approach. Finally, the ligands’ physicochemical characteristics were evaluated using the Free ADME-Tox Filtering Tool (FAFDrugs4) webserver [52] and MOE [49].

#### 2.1.3. Validation of Docking and Virtual Screening Protocols

Benchmark studies using the GOLD v5.07 software package were conducted to validate the docking protocol [53]. Four scoring functions, ChemPLP [54], GoldScore [55], ChemScore [53], and ASP [56], were evaluated. Prior to each ligand being docked against the available protein crystal structures, independent docking simulations were run for each ligand, scoring function, and reference receptor docking residue.

The docking binding site center was anchored using Lys84, Asn36, and Asp862 residues. These key residues were identified through visual inspection and using CAVIAR (CAVity Identification and Rationalization), an open-source tool designed to generate descriptors for binding sites and identify protein cavities using 3D structures [57]. The docking region was defined with a 15 Å radius around these residues. All scoring functions were assessed for their efficacy in scoring and ranking, with ChemPLP ultimately chosen for subsequent virtual screening campaigns, utilizing 500 GA runs and centering on the Lys84 residue.

For virtual screening calculations, the ChemBridge database [58], which encompasses approximately 2 million compounds, was utilized. Selected compounds from this collection were purchased for further studies. The docking-based virtual screening produced compounds ranked based on multiple criteria, including scoring metrics, ligand efficiency, binding pocket fit, and interactions with adjacent residues.

Molecular weight, hydrogen-bond donors and acceptors, rotatable bonds, and logP values were the main topics of the comprehensive investigation. Using the MOE software, compounds that target the Lys84 residue pocket in XPG were visually examined. The specific position of top-ranking molecules within the binding pocket was prioritized. This involved a detailed analysis of how well each molecule fit within the defined cavity, considering spatial orientation and interactions with key amino acid residues. These interactions, including hydrogen bonds, hydrophobic contacts, and π-stacking, were meticulously evaluated to ensure the molecules were optimally posed for potential inhibitory activity. Compounds exhibiting “Pan Assay Interference Compounds” (PAINS) scaffolds were systematically evaluated and eliminated to ensure reliability and specificity in potential inhibitory activity.

#### 2.1.4. Key Interactions, Chemical Space, and Clustering of Structurally Similar Scaffolds

The Murcko Scaffold decomposition method within RDKit 2020.09.1 software [59] was employed to cluster structurally similar scaffolds. Scaffold similarity was assessed by calculating the Morgan fingerprints and the Tanimoto coefficient, which are also included in RDKit. Both 2D and 3D physicochemical descriptors for these scaffolds were calculated using RDKit. To visualize the clustering of structures, t-distributed Stochastic Neighbor Embedding (t-SNE) was applied for dimensionality reduction, projecting the original 1024-dimensional data into a 2D representation using the t-SNE function in the scikit-learn Python module version 3.7.13 [60].

Protein–ligand interactions within PDB files were examined and visualized, facilitating a comparative assessment of interactions across different systems. Various interactions were considered in the analysis, including hydrogen bonds, hydrophobic interactions, π-stacking, water bridges, halogen bonds, and salt bridges. This comprehensive interaction profiling provided valuable insights into the binding characteristics and potential efficacy of the compounds studied. The Protein–Ligand Interaction Profiler (PLIP) algorithm was used for each system under study, utilizing the PDB files and the PLIP Python module [61].

### 2.2. Cell-Based Assays

#### 2.2.1. Chemicals

RPMI-1640 medium supplemented with L-glutamine and HEPES were procured from Biowest (Nuaillé, France), while trypsin (0.25%), Penicillin–streptomycin solution (10,000 units/mL of penicillin; 10 mg/mL of streptomycin), and fetal bovine serum (FBS) were obtained from Gibco (Paisley, UK). Cisplatin and crystal violet (CV) were gathered from Sigma-Aldrich (Madrid, Spain), and sodium chloride from Labchem (Lisboa, Portugal). Dimethyl-sulfoxide (DMSO) was acquired from Fisher Chemical (Waltham, MA, USA), and sodium pyruvate was purchased from PAN Biotech (Aidenbach, Germany). Sodium bicarbonate and D-Glucose were sourced from AppliChem (Darmstadt, Germany).

Cisplatin was prepared in saline at 2 mM (stock solution) and stored at −20 °C. Compounds acquired from the ChemBridge database were reconstituted in 100% DMSO and applied to cell-based assays, as reported by Manguinhas et al. [39]. Solvent-treated controls were also included in all in vitro assays, i.e., cells were exposed to DMSO (0.4% (*v*/*v*)) and/or saline.

#### 2.2.2. Cell Culture

Human NSCLC cell lines H1975, A549, H460, H1993, HCC827, H1299, and the human bronchial epithelial non-cancerous cell line BEAS-2B were obtained from the American Type Culture Collection (ATCC, Manassas, VA, USA). They were maintained in monolayer in RPMI-1640 medium supplemented with L-glutamine, 10 mM HEPES, 2.5 g/L D-glucose, 1 mM sodium pyruvate, 1.5 g/L sodium bicarbonate, 10% FBS, and 1% penicillin/streptomycin, at 37 °C in a humidified atmosphere with 5% CO_2_.

#### 2.2.3. Gene Expression

To identify the most suitable NSCLC cell model for investigating potential XPG inhibitors, *ERCC5* (XPG) expression was analyzed in all cell lines using quantitative reverse transcription PCR (qRT-PCR), as described in Manguinhas et al. [39]. The following primer sequences were utilized: *ERCC5* (XPG) (forward: 5′-GCATGAAATCTTGACTGATATGAAAGA-3′, reverse: 5′-TAAGCAAGCCTTTGAGTTGGTACTG-3′); *β-actin* (forward: 5′-CATGTACGTTGCTATCCAGGC-3′, reverse: 5′-CTCCTTAATGTCACGCACGAT-3′). The relative expression levels of the *ERCC5* (XPG) gene in each cell line were calculated using the 2^−∆Ct^ method, where ∆Ct represents the difference between the average Ct values of the gene of interest and the reference gene *β-ACTIN*. Duplicate samples of cDNAs were analyzed and three independent samples were collected.

#### 2.2.4. First Screening Approach for the XPG Inhibitors in a NSCLC Cell Line

The XPG putative inhibitors were initially subjected to combinatory cell-based experiments with cisplatin. Each compound (50 μM) was tested in combination with cisplatin (1 μM) and applied to H1299 cells, which exhibited higher *ERCC5* expression. The 3-(4,5-dimethylthiazol-2-yl)-5-(3-carboxymethoxyphenyl)-2-(4-sulfophenyl)-2H-tetrazolium salt (MTS) reduction assay was used as a measure of cell viability, as previously outlined by Manguinhas et al. [39], after a 72-h incubation period with both compounds. The absorbance values exhibited by control cells were considered as 100% cell viability. Two to three independent experiments were performed, and three replicates were used for each condition in each independent experiment.

For those compounds exhibiting significant cytotoxicity in the initial screening, a subsequent screening assay was conducted in the same cell line, with their concentration reduced to 10 µM. The same experimental conditions were followed. This procedure was repeated across two to three independent experiments.

#### 2.2.5. Pre-Clinical Safety Assessment in BEAS-2B Cell Line

A pre-clinical safety assessment in non-malignant cells was performed by treating the human bronchial epithelial BEAS-2B cell line with the most promising compounds (enhancing cisplatin-induced cytotoxicity). The MTS assay was also used herein as the cell viability endpoint with the same protocol as mentioned above. Each compound was applied at concentrations of 50 µM for CB9-G, CB20-20, CB22-G, CB23-G, CB41-G, and CB49-G; 25 µM for CB46-G; and 10 µM for CB60-G for 72 h. Solvent-treated control cells represented 100% of cell viability. Two–three independent experiments were achieved with three replicates for each condition.

As a complementary endpoint of cell viability, the crystal violet (CV) staining assay was also performed in the BEAS-2B cell line. The exact same conditions were applied as in the previous MTS assay for each compound. The protocol for this assay was performed as described in Manguinhas et al. [39,62]. Solvent-treated control cells represented 100% of cell viability. Three independent experiments were achieved with six replicates for each condition.

#### 2.2.6. Statistical Analysis

The results are presented as Average ± SD and were analyzed with one-way ANOVA multiple comparisons with GraphPad Prism^®^ 9.0. *p* < 0.05 was considered statistically significant (represented as: * *p* < 0.05, ** *p* < 0.01, *** *p* < 0.001, and **** *p* < 0.0001).

## 3. Results and Discussion

### 3.1. Computational Studies for Targeting the XPG Protein: Drug Development Protocol

#### 3.1.1. Collection of Reported Molecules Tested for XPG Activity

A group of 64 molecules studied for their inhibitory activity towards XPG was compiled from the ChEMBL28 database [51]. This collection was chosen to validate the docking protocol to be used in the virtual screening calculations. The molecules obtained from ChEMBL exhibited a wide range of inhibitory activities towards XPG and were reported in two previous studies specifically focused on the discovery of Flap endonuclease-1 (FEN1) inhibitors [63,64]. In those studies, the XPG activity was also evaluated to check for enzyme specificity. The molecules that were also identified to display activity against XPG were primary derived from 2,4-diketobutyric acid and 3,4-diketobutyrates, with various substitutions. These compounds were used to validate the molecular docking protocol (as described in Section 2.1) for the virtual screening campaign described below.

A thorough investigation was carried out, with a particular emphasis on various properties and descriptors, such as molecular weight, logP, H-bond acceptors and donors, and Lipinski violations, among others. Molecular descriptors were calculated using FAFDrugs4 [52] and MOE [49], which include both conventional and pharmacophore-based descriptors matched with drug-likeness criteria, ideal drug attributes, and medicinal chemistry properties. The structural diversity of these molecules was significant, with molecular weights ranging from less than 200 g/mol to 412 g/mol and a wide range of logP values, reflecting their different lipophilicity. The logP values in the dataset were up to 4, and all molecules adhered to the Lipinski rule of 5.

**Figure 1:**
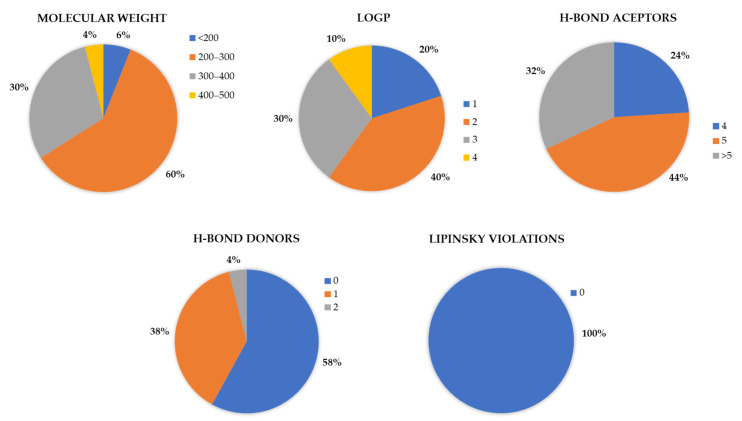
Graphical representation of the variance in the molecular descriptors of molecules retrieved from ChEMBL with activities ranging from 23 nM to 30 µM. The assessed descriptors include molecular weight, Lipinski violations, H-bond donors, H-bond acceptors, and logP. Out of a total of 64 molecules, 50 were used for these graphical analyses.

When examining the dataset of compounds with activities up to 30 µM, defined as active from the ChEMBL dataset, it was observed that the logP values predominantly ranged between 1 and 3 (1.5–2.5 more exactly), and the molecular weights primarily fell between 200 to 300 g/mol, with 0 to 1 H-bond donors and 4 or more H-bond acceptors, without any violations of Lipinski’s rule of 5 (Figure 1). This comprehensive analysis, which presents features indicating a good drug-like profile focused on Lipinski’s rules, is crucial for selecting compounds after virtual screening, offering a foundation for further biological assays and enhancing our understanding of the molecular characteristics that influence XPG inhibition.

#### 3.1.2. Selection and Preparation of XPG Structures

XPG plays a pivotal role in DNA damage repair via the NER pathway. Structurally, XPG is characterized by the N- and C-subdomains, and a helix-2turn-helix domain (H2TH) that forms the K^+^ binding site and β-pin, which is responsible for DNA interaction [48]. As an endonuclease, XPG belongs to a superfamily with a conserved catalytic domain comprising two regions, “N” and “I”. What sets XPG apart from other endonucleases is the insertion of 680 amino acids, known as the R-domain or spacer region, and an extended C terminus after the “I” region. These insertions interfere with the crystallization process due to predicted coiled coils and disordered regions [47]. Available PDB structures support this observation, showing gaps and requiring careful selection for docking studies. The 3D structure of XPG features a core with a higher presence of β-sheets, a seven-stranded twisted β-sheet center, and α-helices connecting different regions, impacting both the DNA binding region and the active site. The active site is characterized by a high number of carboxylates, connecting the N- and I-subdomains and involving the spacer region, which functions as flexible linker to facilitate the native configuration of the structure [48].

To select adequate XPG structures, a systematic search was performed in PDB, retrieving a total of seven 3D structures (Table 1). Of these, only five were of human origin. The non-human structures were discarded, and the remaining ones were categorized into apo structures and XPG–DNA complexes. It is also important to mention that these five structures were resolved very recently in 2020, showing that this target is gaining the attention of the scientific community.

The structural characterization of XPG has provided insights into its role in DNA repair. High-resolution crystallography has elucidated the complex architecture of XPG, highlighting the importance of its endonuclease and DNA-binding domains, interdomain linkers, and protein–protein interaction regions. Understanding these structural features is crucial for deciphering the molecular mechanisms underlying XPG’s function and developing therapeutic strategies targeting its function.

**Figure 2:**
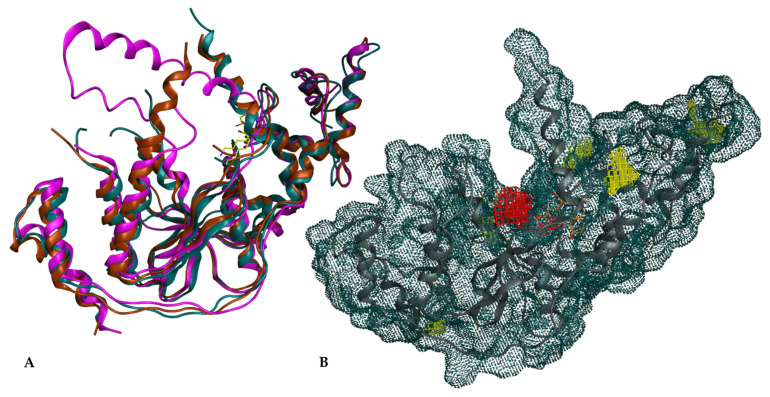
XPG structure and surface representation. (**A**) Overlap of XPG structures from PDB IDs: 6TUR (turquoise), 6TUX (maroon), 6VHB (purple) and Lys84 of each structure (yellow); (**B**) Molecular surface representation of XPG, tinted in turquoise, highlighting the active site in red and alternative cavities in yellow.

Like other endonucleases, XPG has the potential to bind to DNA via its active site, which was the primary focus of this study. The active site forms an access point between the helices, making it a critical region for targeting [47]. For the docking studies, the cavity around Lys84 was selected as the binding site due to its high conservation across the XPG superfamily and its ordered positioning (Figure 2A). Structural analysis indicated that the residue Lys84 is crucial for maintaining XPG activity, since modifying this residue could inhibit the endonuclease function [48]. Lys84 is strategically positioned in a central cavity formed by the protein, making it an ideal target for docking calculations, now referred to as the Lys84 cavity (red surface in Figure 2B).

The region defined by the Lys84 cavity is conserved across all available 3D structures of XPG, with the proximity zone of this residue being best preserved in the 6TUR structure. No previous computational studies targeting this protein have been reported, and no crystallographic structure of XPG complexed with an inhibitor is available. This gap underscores the novelty and potential impact of research in identifying inhibitors for XPG.

In the initial analysis, the retrieved 3D structures of XPG proteins (Table 1) were aligned and superimposed. Figure 2A shows the overlap of the 6TUR, 6TUX and 6VHB structures. The root mean square deviations (RMSDs) of the structures ranged from 0.67 Å to 1.93 Å, indicating high structural similarity. The overlap revealed significant resemblance, particularly around the putative binding pocket region defined around Lys84, as identified using MOE [49]. The pocket surrounding Lys84 is highly conserved, suggesting its potential as a key site for inhibiting endonuclease activity. In the 6VHB structure, the RMSD spiked to 3.97 Å, and more gaps were observed.

**Figure 3:**
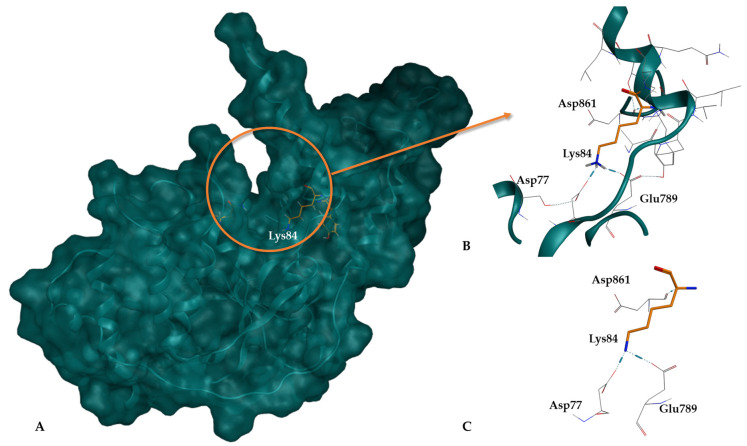
XPG protein structure (6TUR). (**A**) Representation of the XPG 6TUR protein surface in blue, highlighting the location of the binding pocket; (**B**) Region of the XPG structure with emphasis on the pocket cavity and residue Lys84; (**C**) Close-up of Lys84 (in orange) at the pocket entrance, showing its interaction with nearby residues.

When comparing the two apo structures, 6TUR and 6TUS, it was found that in 6TUR, Lys84 is positioned deeply within the pocket, which is advantageous for docking calculations (Figure 3). In this structure, Lys84 interacts with Asp77, Glu789, and Asp861, forming H-bond interactions. Conversely, in 6TUS, Lys84 is near a gap in the structure (with residue 92 absent), which could mask the 3D conformation, leading to its exclusion.

In structures 6TUX and 6TUW, where XPG is linked to a DNA fragment, structural rearrangements occur due to the binding. These changes justified the decision to proceed with the apo structure for further studies. Using an apo receptor is rationalized by the hypothesis that interference with the DNA repair mechanism can be better understood without DNA-induced conformational changes. The absence of residue 92 in 6TUS further supports the use of the 6TUR apo form for accurate analysis. Therefore, the apo structure 6TUR was selected to mimic the stage before DNA interaction, favoring inhibition and disruption of the repair mechanisms.

An independent analysis using CAVIAR confirmed the selected binding pocket around Lys84 as the highest-scoring cavity with a size of 280 Å^3^, a hydropathicity of 36%, and a drug binding site classification of 0.6/1 (Figure 2B) [57]. This classification supports the high potential of this cavity as a drug binding site. CAVIAR identified a total of six potential cavities in the XPG structure (cavities colored yellow in Figure 2B).

#### 3.1.3. Selection of the Docking Protocol

After selecting the dataset of small molecules and the receptor structure, docking calculations were performed to validate the protocol for virtual screening. The docking search space was defined to encompass the interaction region around the Lys84 residue. Lys84 was tested as the center of the search space, along with two additional residues that were also evaluated for their potential as binding pocket centers. Comprehensive analyses and comparisons were conducted on all results from these molecular docking calculations to identify and validate the most accurate point that best reproduces the available experimental data. In addition, four well-known scoring functions—ChemPLP, GoldScore, ChemScore, and ASP—available in the GOLD v5.07 software suite were evaluated. In this validation process, the previously prepared ChEMBL dataset (see Section 2.1) was docked into the selected XPG 3D structure within a 15 Å radius of the identified binding site centers. The docking site center was anchored using the residues Lys84, Asn36, and Asp862, located at the bottom of the binding cavity (Figure 3). Following the completion of the docking calculations, the results were analyzed to evaluate any correlations between each molecule’s score and its experimental inhibitory activity. The Lys84 residue demonstrated the best reproduction of molecular interactions in the binding site and showed the best correlation with experimental inhibitory activities. A similar procedure was followed for the four scoring functions. The main goal of this process was to improve the protocol’s ability to identify highly active compounds and provide more accurate scoring. To the best of our knowledge, no previous protocol was available for reference. Although the docking scores were not particularly high, the molecules were well-positioned within the pocket defined by the Lys84 residue. As mentioned earlier, the docking cavity is slightly open which we hypothesize may influence the score attributed by GOLD.

The best and most consistent parameters were found by taking into account all of the molecular docking results for the 64 ChEMBL compounds. These included using 500 GA runs and the GOLD v5.07 program with the ChemPLP score function. Docking was centered on the Lys84 residue in the XPG protein, utilizing the 6TUR crystallographic structure. This combination resulted in the best placement of molecules within the pocket and the highest correlation between docking scores and experimental inhibitory activity across all tested systems.

#### 3.1.4. Virtual Screening Campaign

**Figure 4:**
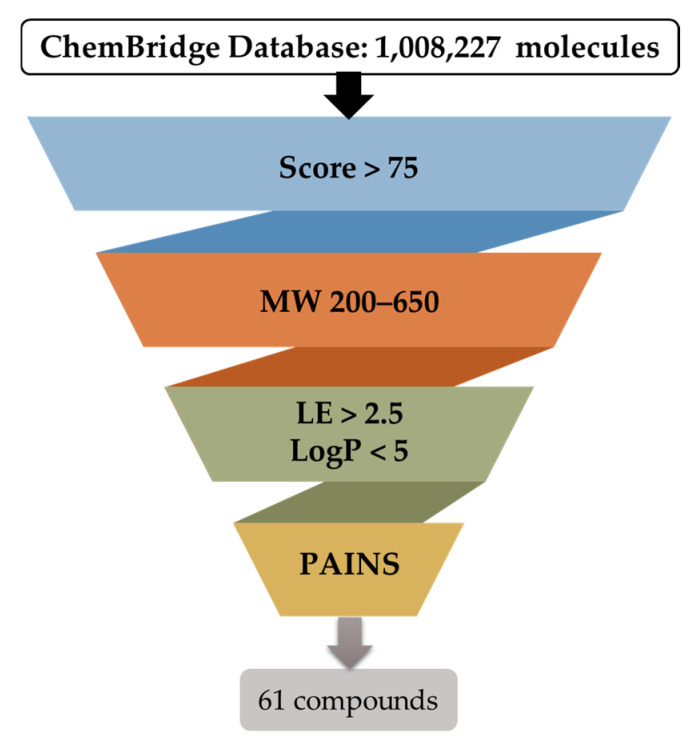
Schematic representation of the filtering criteria applied for compound selection from the ChemBridge database. A total of 1,008,227 molecules were subjected to docking (individually) and scored to find the top 5000 molecules. They were then selected for further filtration using the specified criteria and, consequently, 61 compounds were proposed for acquisition.

Following the establishment of the docking protocol, the virtual screening campaign (Figure 4) was initiated using the ChemBridge library, which includes a diverse collection of 1,008,227 compounds. The strategic selection of ChemBridge aimed to maximize the chances of identifying novel and potent XPG inhibitors (hits) by comprehensively exploring a chemical space that encompassed a variety of functional groups, scaffolds, and stereochemical configurations.

According to the optimal docking protocol identified earlier in this study, docking-based virtual screening calculations were conducted using GOLD software with the ChemPLP fitness scoring function, 500 GA runs, and Lys84 as the docking site center within a 15 Å radius. The docking results were filtered based on several criteria: docking score, molecular weight, ligand efficiency, logP, and PAINS scaffolds (Figure 4). Visual assessment was also crucial for selecting hits to ensure proper fitting within the binding site region. These filter cut-offs and ranges were defined based on the evaluation of XPG inhibitors retrieved from the ChEMBL database (Figure 1) and established drug-like rules. The analysis revealed that, for active compounds, a cut-off score of 75 was appropriate, with molecular weights ranging between 200 and 650 Da, and logP less than 5. The restrictions of molecular weight and logP were expanded beyond the initial dataset to evaluate the possibility of finding compounds with more diverse scaffolds. The compounds previously reported were from the same dataset, so it can be more limitative in terms of synthetized molecules. By enlarging the limits of these two properties while maintaining appropriate drug-like values, more molecules are allowed to be analyzed through virtual screening.

After applying the filters described in Figure 4, a visual inspection of 316 compounds was performed, reducing the identification to 61 hit compounds. Visual assessment was crucial to ensure proper fitting within the region. The docking site was not the typical defined cavity with only one point of access, so reducing the hits dataset to a manageable number for visual inspection was essential. The 316 molecules were then subsequently clustered based on structural similarities to ensure maximum scaffold diversity in the final dataset.

**Figure 5:**
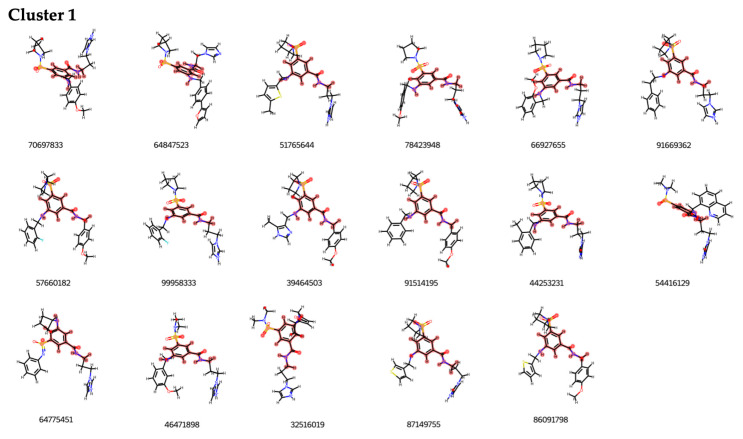
Representation of Cluster 1 derived from the ChemBridge results dataset identified through visual inspection. Molecule segments highlighted in red denote the shared scaffold that characterizes each cluster.

The clustering process applied revealed 48 distinct clusters. Clusters 1 and 2, which featured the most prevalent scaffolds, were the most populated, each containing 17 and 13 structures, respectively (see Figure 5). This analysis highlighted the presence of different substituents on a shared scaffold within each cluster. The high number of clusters, each with a relatively small number of molecules, underscores the molecular diversity among potential XPG inhibitors. Each cluster’s molecules were visually inspected to determine their positioning within the binding pocket, which then guided the selection of compounds.

By examining the ranked list of the 316 molecules by their properties, it was observed that, despite the high number of clusters, the top-classified molecules did not share the same clusters. A significant number of molecular clusters with only two molecules (18 out of 48) were identified, explaining the dispersion of scaffolds between these groups. The assessment, incorporating visual inspection and clustering of structurally similar scaffolds through the Murcko Scaffold decomposition method in RDKit, evaluated similarity and refined the selection of molecules for this challenging target.

**Figure 6:**
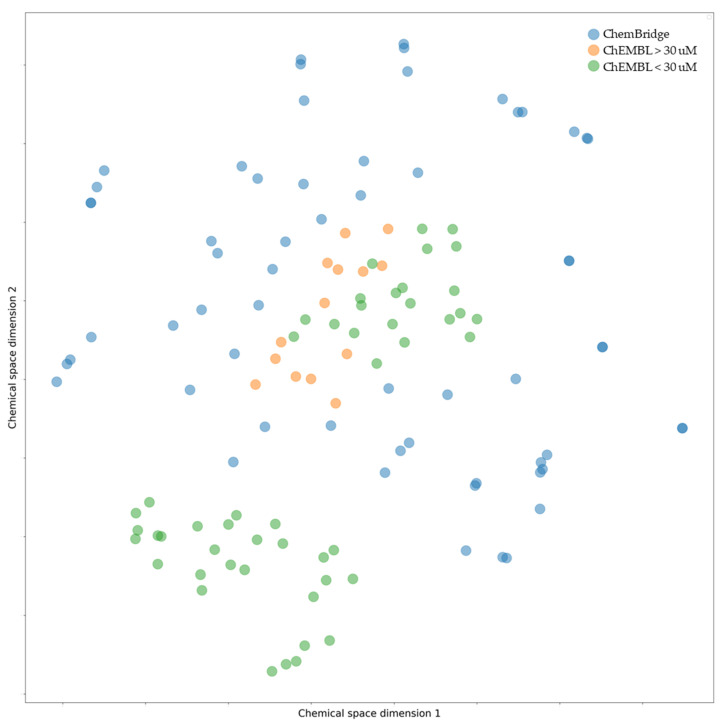
t-SNE plot illustrating the chemical diversity of active (green) and inactive (orange) compounds from ChEMBL. Additionally, hit molecules from the ChemBridge database are shown in blue. Active compounds display a wider range of chemical diversity compared to inactive compounds.

This drug design protocol’s primary purpose was to find compounds characterized by a broader and diverse chemical profile than those listed in earlier studies. The chemical space [60] of the compounds that were ultimately selected was defined and shown in Figure 6 in order to evaluate molecular diversity. This analysis juxtaposed the chemical characteristics of compounds from the ChEMBL database, which either exhibited activities above or below 30 nM, with those selected from the ChemBridge dataset. Compounds identified by our virtual screening campaign are more widely spread across the chemical space compared to the narrowly clustered compounds from ChEMBL, which tend to cluster into two main groups.

The rationale for this clustering in ChEMBL is due to the fact that they were originated from a single research group. In contrast, the virtual screening protocol employed here revealed structures free from the restrictive structural constraints tied to previously tested compounds, adhering strictly to the Lipinski’s rule of five, so promoting a more diverse chemical repository. This methodological approach facilitated the discovery of a wide range of novel compounds.

**Figure 7:**
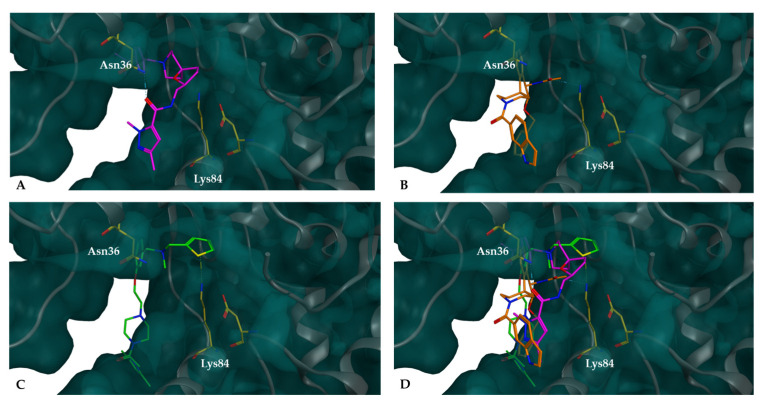
Compounds docked into the binding pocket of the XPG protein are represented as follows: XPG highlighting the Lys84 and Asn36 residues inside the binding pocket; (**A**) docked complexes of XPG with potential inhibitor CB41-G (purple), (**B**) docked complexes of XPG with potential inhibitor CB60-G (orange), (**C**) docked complexes of XPG with potential inhibitor CB22-G (green), and (**D**) overlap of the three small molecules. All these molecules fit snugly within the pocket, effectively blocking access to the top of the cavity.

In Figure 7, the potential inhibitors selected from the ChemBridge database are compared in terms of their position within the binding site relative to the Lys84 residue of the XPG pocket. The compounds either participated in molecular interactions with the Lys84 residue or were positioned close to it. For example, CB41-G interacts with Asn36, and its exposure to the pocket revealed the importance of the placement of the small molecules. All three examples demonstrated access to the cavity in different directions while keeping access to the internal region of the pocket. The aromatic rings of the compounds were positioned on the outer side of the pocket, whereas the tertiary alkyl amine group was oriented towards the inner region of the pocket, with different substituents. It is noteworthy that the Lys84 residue also displays an alkyl amine group side chain in this same region.

### 3.2. Validation of the Proposed XPG Protein Inhibitors in Cell-Based Assays

#### 3.2.1. Characterization of ERCC5 Expression Levels in NSCLC Cell Lines

**Figure 8:**
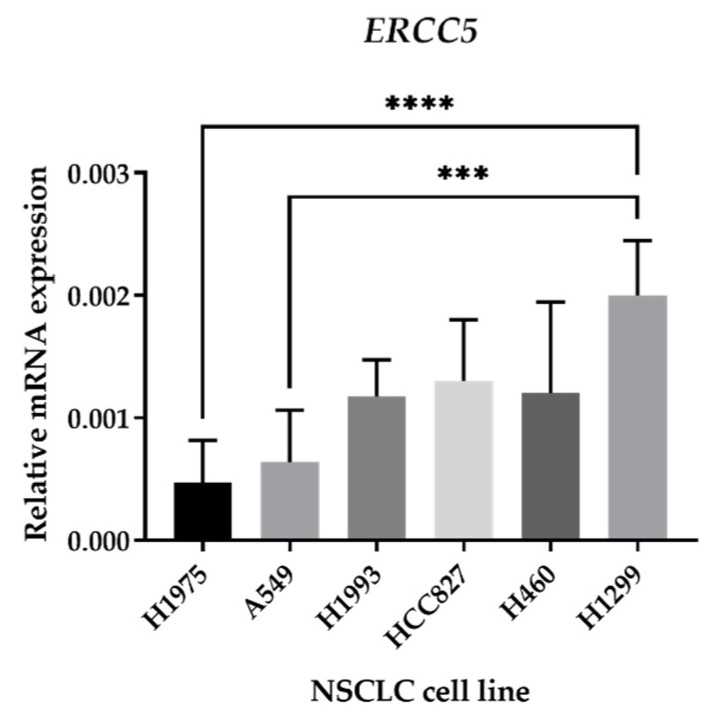
mRNA expression of the *ERCC5* gene in NSCLC cell lines. qRT–qPCR was used to analyze *ERCC5* expression of all six NSCLC cell lines using isolated RNA samples. Relative expression was obtained by calculating 2^−∆Ct^, where ∆Ct = Average Ct (gene of interest)—Average Ct (*β-ACTIN*) and normalized it to *β-ACTIN*. The results are presented as mean ± SD (*n* = 3). *** *p* < 0.001 and **** *p* < 0.0001 (one-way ANOVA, Tukey’s multiple comparisons test).

To select the best NSCLC cellular model for the validation of XPG putative inhibitors, mRNA levels of the *ERCC5* gene were assessed using qRT-PCR. The ERCC5 gene codes for the XPG protein. A total of six NSCLC cell lines were evaluated. The results indicated the H1299 cell line has the highest *ERCC5* expression levels, followed by the H1993, HCC827, and H460 cell lines. H1975 and A549 cells have the lowest *ERCC5* levels, respectively (Figure 8). Based on these results, the H1299 cell line was selected for further cell-based assays. This selection was relied on the basis that cell lines expressing higher levels of *ERCC5* would better mimic a clinical situation where tumors are overexpressing this NER component.

#### 3.2.2. First Screening Approach to Assess the Impact of the Proposed Inhibitors in Cisplatin-Induced Cytotoxicity

**Figure 9:**
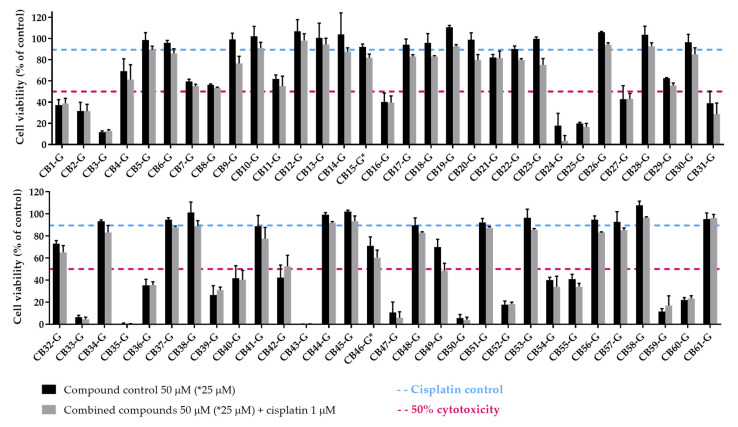
Sensitizing effect of the acquired XPG inhibitors on cisplatin-induced cytotoxicity. The MTS assay was performed as a measure of cell viability and all the compounds (50 µM) selected from the ChemBridge database were applied to H1299 cells in combination with cisplatin (1 µM) for 72 h. Compounds marked with “*” were tested at 25 µM due to solubility issues. Values represent mean ± SD and are expressed as percentages of the solvent-treated control cells that were considered as 100% of cell viability (*n* = 2–3).

After the selection of the most suitable cellular model and acquisition of the proposed XPG inhibitors, a first screening approach was performed by assessing if the inhibitors could enhance cisplatin-induced cytotoxicity. A similar methodological approach was conducted as described in Manguinhas et al. [39]. An MTS reduction assay was also selected to measure cell viability in the H1299 cell line after exposure to 1 µM of Cisplatin and 50 µM of each putative inhibitor for 72 h (25 µM for CB16-G and CB46-G inhibitors due to solubility issues). Figure 9 presents the results for all putative inhibitors selected from the ChemBridge database.

The concentration of 1 µM of cisplatin was applied to induce a moderate decrease in cell viability, of approximately 10–15%, as observed by Manguinhas et al. in H1299 cells [39]. While not causing significant cytotoxicity on its own, this concentration allowed us to clearly observe the enhanced cytotoxic effects when combined with the putative inhibitor. Regarding the putative inhibitors, we selected a concentration of 50 µM, as it represents a balanced compromise between two important considerations: efficacy as NER inhibitors and inherent compound toxicity. Opting for higher concentrations, such as 100 µM, could result in increased cytotoxicity due to the compounds’ intrinsic cytotoxic properties. Conversely, reducing the concentration during the initial screening phase might lead to overlooking potentially effective compounds that only demonstrate activity at higher concentrations.

**Figure 10:**
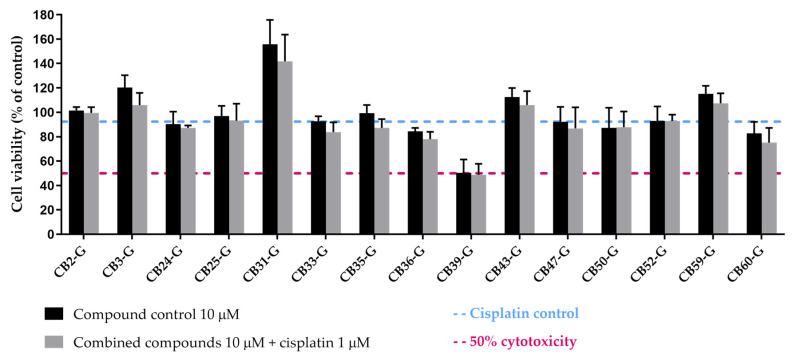
Effect of the acquired XPG inhibitors, which were considered extremely cytotoxic at 50 µM, on cisplatin-induced cytotoxicity. The MTS was performed in H1299 cells after 72 h exposure to each compound (10 µM) and cisplatin (1 µM). Values are presented as mean ± SD and were calculated as percentages of the solvent-treated control cells (100% cell viability, *n* = 2–3).

**Figure 11:**
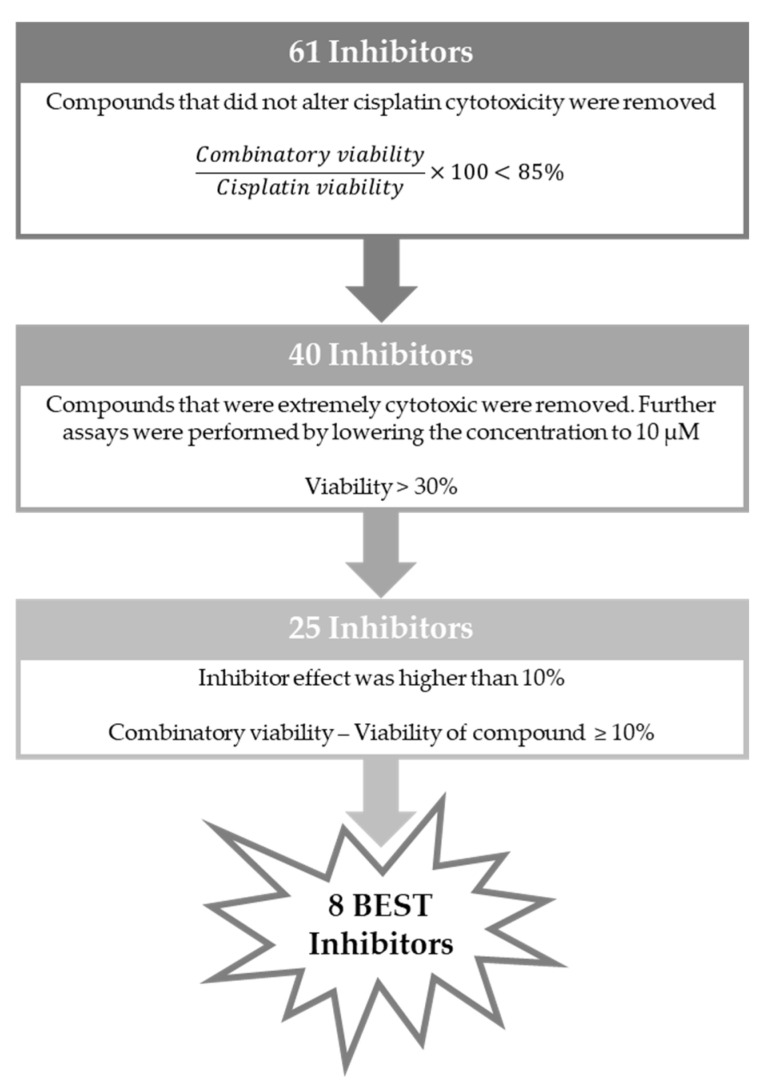
Strategy employed to select the 8 best inhibitors.

To select the most promising XPG inhibitors, a decision-making process was established, similar to that in our previous study [39]. Briefly, the first step of selection was based on the effect of cisplatin combination with each inhibitor towards cisplatin alone. If cell viability of the combined condition relative to cisplatin alone was less than 85%, indicating an increase in cisplatin cytotoxicity of at least 15%, the compounds were selected to the next stage. Consequently, 21 compounds were removed from further consideration. The next step of exclusion was based on each compound intrinsic cytotoxicity. Those causing over 70% loss in cell viability at 50 µM (25 µM for CB16-G and CB46-G) were removed from consideration. However, they were not discarded at this phase but rather subjected to further investigation at a lower concentration (10 µM—see Figure 10). This step resulted in the exclusion of 15 compounds from the pool of top candidates. From the remaining 25 compounds, the last feature for selection was based on the direct effect of the inhibitor on the combinatory effect, independent of its own cytotoxicity in the cells. Hence, the inhibitor’s viability was subtracted from the viability of the combination of both compounds. Compounds with an effect greater than 10% were considered among the best inhibitors. Consequently, seven compounds emerged as the best candidates for further assessment: CB9-G, CB20-G, CB22-G, CB23-G, CB41-G, CB46-G, and CB49-G. The process for selection is depicted in Figure 11.

From the screening at 10 µM of the extremely cytotoxic 15 compounds (Figure 10), only CB60-G emerged as a promising compound to boost cisplatin cytotoxicity. Based on this assessment, CB60-G was evaluated with the same criteria proposed in Figure 11, and later selected among the pool of best inhibitors, totaling eight best inhibitors for further evaluation. The chemical structures of the eight best inhibitors are present in Supplementary Materials, Figure S1.

#### 3.2.3. Validation of the Identified XPG Inhibitors and Evaluation in a Non-Tumoral Cell Line

**Figure 12:**
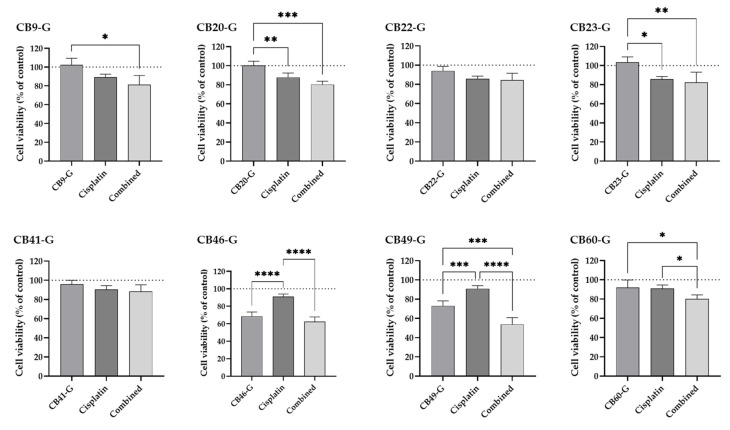
Effect of the 8 best inhibitors in sensitizing H1299 cells to cisplatin-induced cytotoxicity. The MTS assays was performed as a measure of cell viability and all the compounds (CB9-G, CB20-20, CB22-G, CB23-G, CB41-G, CB49-G at 50 µM; CB46-G at 25 µM; CB60-G at 10 µM) were applied to the cells in combination with cisplatin (1 µM) for 72 h. Values represent mean ± SD and are expressed as percentages of the solvent-treated control cells considered as 100% of cell viability, identified as the dotted line in the graphs (*n* = 3–5). * *p* < 0.05, ** *p* < 0.01, *** *p* < 0.001, and **** *p* < 0.0001 (one-way ANOVA, Tukey’s multiple comparisons test).

The eight compounds selected as best inhibitors were further assessed using the cell viability MTS assay. The same experimental conditions were applied as in the previous first screening approach and cisplatin was tested at 1 µM alone and in combination. The compounds CB9-G, CB20-20, CB22-G, CB23-G, CB41-G, and CB49-G were incubated at 50 µM, CB46-G at 25 µM, and CB60-G at 10 µM. Our main goal was to compare the effect of each inhibitor in cisplatin-induced cytotoxicity (Figure 12). Overall, CB9-G, CB20-G, B22-G, CB23-G, CB41-G and CB60-G demonstrated to have no cytotoxicity at the concentrations tested (not statistically significant when compared to the control). Only CB46-G and CB49-B exhibited a cytotoxicity of around 30% to H1299 cells (*p* < 0.05). In terms of cisplatin effect alone, loss in cell viability was around 10–15%. When taking into consideration the compounds that indeed had a significant effect in cisplatin-induced cytotoxicity (loss of more than 15% in cell viability), only CB46-G, CB49-G and CB60-G demonstrated to have a sensitizing effect. In fact, CB46-G was able to enhance cisplatin-induced cytotoxicity in 31%, CB49-G in 41%, and CB60-G in 12%.

**Figure 13:**
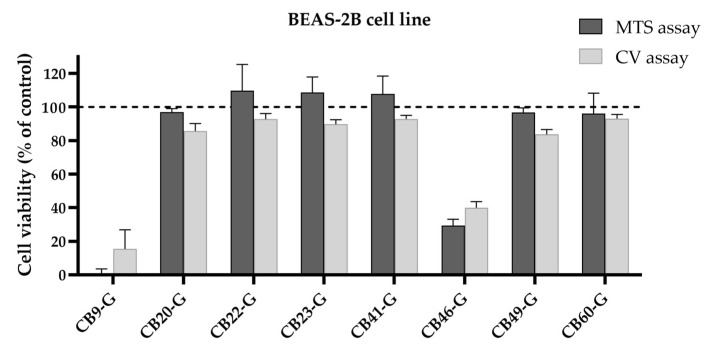
Cytotoxicity of the 8 best inhibitors in BEAS-2B cells. Cell viability was assessed in the BEAS-2B cell line using the MTS reduction and the CV staining assays by incubating each compound for 72 h (CB9-G, CB20-20, CB22-G, CB23-G, CB41-G, CB49-G at 50 µM; CB46-G at 25 µM; CB60-G at 10 µM). Values represent mean ± SD and are expressed as percentages of the solvent-treated control cells considered as 100% of cell viability, identified as the dashed line in the graph (*n* = 2–3).

As a pre-clinical safety assessment, we tested the eight best inhibitors in a non-tumoral human bronchial epithelial cell line (BEAS-2B). Two complementary cell viability approaches were considered, the MTS reduction and the CV staining assays. Each compound was incubated with the cells for 72 h at the same concentrations used in the precious assays. Based on these results, both cell viability endpoints were in accordance for all the tested inhibitors (see Figure 13). Overall, only CB9-G and CB46-G induced a loss in cell viability of more than 50%. For this reason, CB49-G and CB60-G demonstrated to be promising candidates for use as cisplatin enhancers, with no cytotoxicity for non-tumoral cells.

### 3.3. Interaction Analysis of Inhibitors with XPG Protein

**Figure 14:**
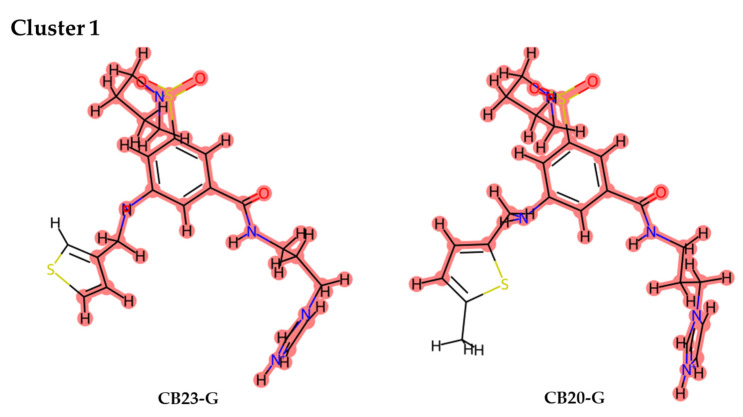
Representation of cluster derived from the final data set of 8 molecules proposed as inhibitors. Between the 8 molecules, only 2 were sufficiently similar to be considered as a cluster. Parts of the molecules highlighted in red represent the similar scaffold that defines each cluster.

From the group of 61 tested compounds, eight were identified as the top inhibitors, prompting a thorough structural examination to clarify any possible interaction mechanisms between these compounds and the XPG protein. Using Murcko Scaffold decomposition in RDKit [59], the clustering of structurally similar scaffolds was conducted. Only one cluster with more than one molecule was identified, reenforcing the structural variability between scaffolds. In CB23-G, the hydrogen atom is substituted by a methyl group to transform into CB20-G, while the rest of the scaffold remains unchanged (Figure 14). Critical molecular interactions between these compounds and the protein involved residues Gly2, Asn36, Gln37, and Arg91, with predominant interactions being hydrogen bonds, hydrophobic interactions, and a π–cation interaction with Arg91. Additionally, CB20-G exhibited hydrophobic interactions with Gln4 and Leu88, which were not identified in CB23-G (Figure 14).

**Figure 15:**
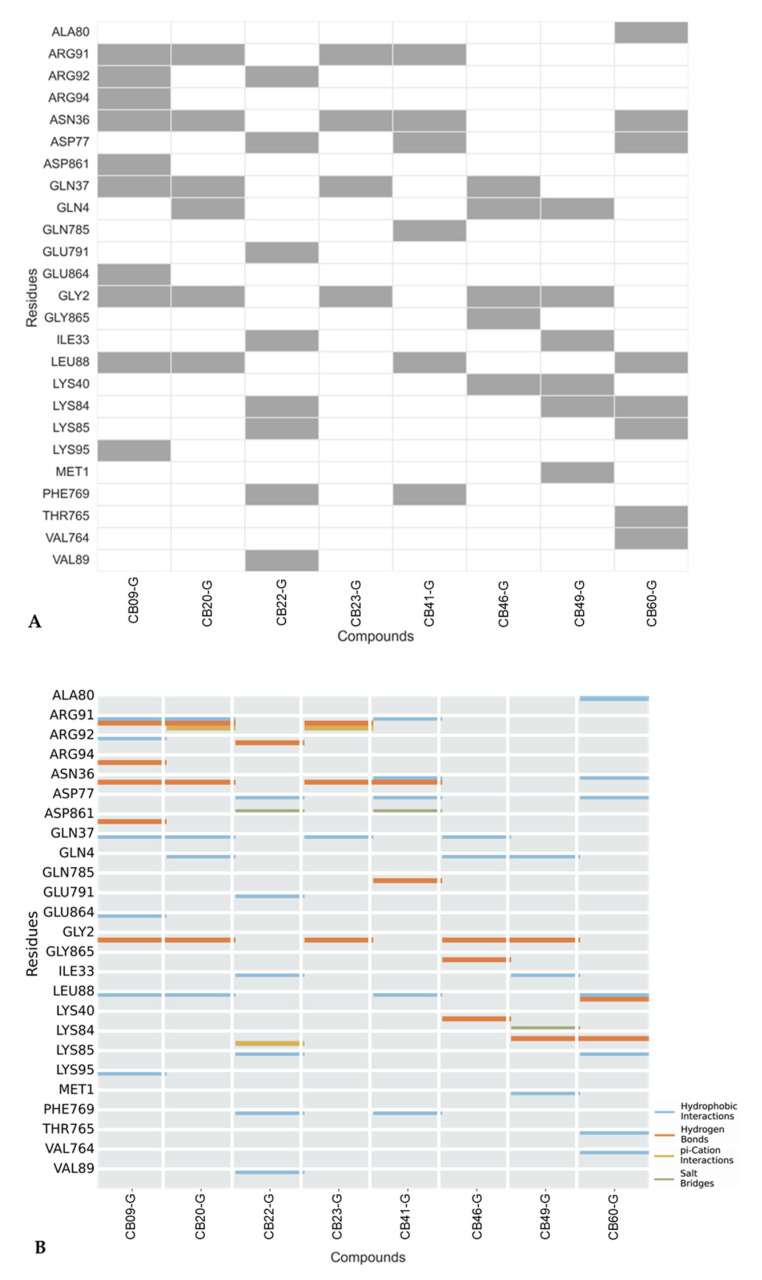
(**A**) XPG residues interacting with the top 8 compounds. Rows represent specific XPG residues, and columns represent active compounds from virtual screening. Grey Squares indicate ligand–receptor interactions, while the white squares indicate no interactions. (**B**) Interaction types between XPG protein and active compounds from virtual screening. Rows represent XPG residues and columns represent the 8 active molecules. The analysis considered H-bonds, hydrophobic interactions, π-stacking, water bridges, halogen bonds, and salt bridges. No water bridges or π-stacking interactions were identified.

Although structurally similar, these molecules interacted with a variety of different residues. (Figure 15A). Analyzing the protein residues involved in the interactions with the small molecules helped to identify key residues that might enhance XPG inhibition when interacting with the eight active compounds in the dataset (Figure 15). Predominantly, hydrogen bonds and hydrophobic interactions were observed between these eight compounds and the XPG residues, as depicted in Figure 15B, with only occasional π-cation interactions and salt bridges. Residues Gly2 and Asn36 interacted with the highest number of compounds, suggesting their important role in potentially promoting XPG inhibition. These residues predominantly engage in hydrogen bonding interactions.

To gain a better understanding of the inhibition correlation between the active and inactive compounds from ChEMBL and the inhibitors proposed in this investigation, key molecular interactions with the XPG protein were evaluated (see Supplementary Materials, Figures S2 and S3). Among the molecules with inhibitory activity greater that 30 µM (14 out of 64), classified as inactive, interactions primarily involved Asn36, Glu789, Glu791, Ile33, and Lys84. Although no distinct interaction pattern signature was identified, Glu 789 and Glu791 interactions were notably prominent among inactive compounds. The type of interactions observed were consistent with the pattern described earlier, as illustrated in Figure 15.

For the molecules with inhibitory activity up to 30 µM, classified as active from ChEMBL dataset, residues such as Asn36, Ile33 and Lys84 interacted with the most compounds, indicating their pivotal role in possibly enhancing XPG inhibition. These residues were also identified in our dataset, with Asn36 being particularly prominent. A significant occurrence of hydrogen bonds and hydrophobic interactions were observed. Lys84 was involved in a major group of interactions and had previously been identified as contributing to better performance in the docking study.

## 4. Conclusions

There is undoubtably a clear need for novel therapeutic strategies to reduce resistance and ameliorate the efficacy of standard drugs currently used to treat NSCLC [65]. This study successfully identified 61 potential XPG inhibitors through an integrated approach combining structure-based virtual screening and molecular docking. Lys84 was identified as a critical residue for XPG activity and targeting this residue with small molecules demonstrated significant potential for inhibiting endonuclease function. The selected inhibitors were validated using the H1299 NSCLC cell model, which has the highest *ERCC5* expression among the tested cell lines. Among these compounds, fifteen exhibited very high cytotoxicity to H1299 cells, and importantly eight emerged as promising candidates for enhancing cisplatin-induced cytotoxicity. Furthermore, the chemical space analysis revealed a broader structural diversity among the selected inhibitors compared to previously reported compounds, indicating that the virtual screening approach effectively explored a wider range of chemical scaffolds. Of the eight compounds identified as promising inhibitors, only three were able to significantly enhance cisplatin-induced cytotoxicity. Additionally, two of these three inhibitors, i.e., CB49-G and CB60-G, presented no cytotoxicity towards the non-tumoral lung cell line (BEAS-2B cells). To conclude, this study proposes CB49-G and CB60-G as the best candidates for XPG inhibition, which should be considered for further studies in order to improve cisplatin therapy in NSCLC patients.

## Figures and Tables

**Table 1:** List of X-ray structures of the XPG protein available in the PDB. This table provides the PDB identifier (PDB ID), resolution (Res.), year of structure determination, structure description, and the corresponding bibliographic reference. The structure highlighted in grey was used for further analysis in this study.

PDB ID	Res. (Å)	Year	Information	Reference
6VBH	2.00	2020	Human XPG endonuclease catalytic domain	[47]
6TUR	2.90	2020	Human XPG, Apo1 form	[48]
6TUS	2.50	2020	Human XPG, Apo2 form	[48]
6TUX	3.10	2020	Human XPG-DNA, Complex 2	[48]
6TUW	3.50	2020	Human XPG-DNA, Complex 1	[48]
5EKF	2.00	2016	Mus musculus XPG complexed with Importin-alpha, fragment 1	[46]
5EKG	2.80	2016	Mus musculus XPG complexed with Importin-alpha, fragment 2	[46]

## Data Availability

Data are contained within the article or Supplementary Materials.

## Supplementary Materials

The following supporting information can be downloaded at: https://www.mdpi.com/article/10.3390/cancers16183174/s1, Figure S1: List and chemical structures of the eight compounds identified as best inhibitors from the ChemBridge database that could enhance cisplatin-induced cytotoxicity; Figure S2: Representation of the types of interactions established between the XPG protein and the compounds retrieved from ChEMBL with activity from 23 nM up to 30 µM (50 compounds); Figure S3: Representation of the types of interactions established between the XPG protein and the compounds from ChEMBL with activity superior to 30 µM (14 compounds).

## References

[1] Chatterjee N., Walker G.C. (2017). Mechanisms of DNA damage, repair and mutagenesis Nimrat. Environ. Mol. Mutagen..

[2] Hakem R. (2008). DNA-damage repair; the good, the bad, and the ugly. EMBO J..

[3] De Bont R., van Larebeke N. (2004). Endogenous DNA damage in humans: A review of quantitative data. Mutagenesis.

[4] Doherty R., Madhusudan S. (2015). DNA repair endonucleases: Physiological roles and potential as drug targets. J. Biomol. Screen..

[5] Rodrigues A., Gomes B.C., Martins C., Gromicho M., Oliveira N.G., Guerreirp P.S., Rueff J. (2013). DNA Repair and Resistance to Cancer Therapy. New Research Directions in DNA Repair.

[6] Huang R., Zhou P.K. (2021). DNA Damage Repair: Historical Perspectives, Mechanistic Pathways and Clinical Translation for Targeted Cancer Therapy.

[7] Helleday T., Petermann E., Lundin C., Hodgson B., Sharma R.A. (2008). DNA repair pathways as targets for cancer therapy. Nat. Rev. Cancer.

[8] Li L.Y., Di Guan Y., Chen X.S., Yang J.M., Cheng Y. (2021). DNA Repair Pathways in Cancer Therapy and Resistance. Front. Pharmacol..

[9] Gavande N.S., Vandervere-Carozza P.S., Hinshaw H.D., Jalal S.I., Sears C.R., Pawelczak K.S., Turchi J.J. (2016). DNA repair targeted therapy: The past or future of cancer treatment?. Pharmacol. Ther..

[10] Rehman F.L., Lord C.J., Ashworth A. (2010). Synthetic lethal approaches to breast cancer therapy. Nat. Rev. Clin. Oncol..

[11] Mesquita K.A., Alabdullah M., Griffin M., Toss M.S., Fatah T.M.A.A., Alblihy A., Moseley P., Chan S.Y.T., Rakha E.A., Madhusudan S. (2019). ERCC1-XPF deficiency is a predictor of olaparib induced synthetic lethality and platinum sensitivity in epithelial ovarian cancers. Gynecol. Oncol..

[12] Mohni K.N., Kavanaugh G.M., Cortez D. (2014). ATR Pathway Inhibition Is Synthetically Lethal in Cancer Cells with ERCC1 Deficiency. Cancer Res..

[13] Topatana W., Juengpanich S., Li S., Cao J., Hu J., Lee J., Suliyanto K., Ma D., Zhang B., Chen M. (2020). Advances in synthetic lethality for cancer therapy: Cellular mechanism and clinical translation. J. Hematol. Oncol..

[14] Beljanski V., Marzilli L.G., Doetsch P.W. (2004). DNA damage-processing pathways involved in the eukaryotic cellular response to anticancer DNA cross-linking drugs. Mol. Pharmacol..

[15] Shuck S.C., Short E.A., Turchi J.J. (2008). Eukaryotic nucleotide excision repair: From understanding mechanisms to influencing biology. Cell Res..

[16] Muniesa-Vargas A., Davó-Martínez C., Ribeiro-Silva C., van der Woude M., Thijssen K.L., Haspels B., Häckes D., Kaynak Ü.U., Kanaar R., Marteijn J.A. (2024). Persistent TFIIH binding to non-excised DNA damage causes cell and developmental failure. Nat. Commun..

[17] Guerreiro P.S., Fernandes A.S., Costa J.G., Castro M., Miranda J.P., Oliveira N.G. (2013). Differential effects of methoxyamine on doxorubicin cytotoxicity and genotoxicity in MDA-MB-231 human breast cancer cells. Mutat. Res.—Genet. Toxicol. Environ. Mutagen..

[18] Kelley M.R., Logsdon D., Fishel M.L. (2014). Targeting DNA repair pathways for cancer treatment: What’s new?. Futur. Oncol..

[19] Barakat K., Tuszynski J. (2012). Nucleotide Excision Repair Inhibitors: Still a Long Way to Go. New Research Directions in DNA Repair.

[20] Gentile F., Tuszynski J.A., Barakat K.H. (2016). New design of nucleotide excision repair (NER) inhibitors for combination cancer therapy. J. Mol. Graph. Model..

[21] McNeil E.M., Astell K.R., Ritchie A.M., Shave S., Houston D.R., Bakrania P., Jones H.M., Khurana P., Wallace C., Chapman T. (2015). Inhibition of the ERCC1-XPF structure-specific endonuclease to overcome cancer chemoresistance. DNA Repair.

[22] McNeil E.M., Melton D.W. (2012). DNA repair endonuclease ERCC1-XPF as a novel therapeutic target to overcome chemoresistance in cancer therapy. Nucleic Acids Res..

[23] Kirschner K., Melton D.W. (2010). Multiple Roles of the ERCC1-XPF Endonuclease in DNA Repair and Resistance to Anticancer Drugs. Anticancer Res..

[24] Muallem M.Z., Braicu I., Nassir M., Richter R., Sehouli J., Arsenic R. (2014). ERCC1 expression as a predictor of resistance to platinum-based chemotherapy in primary ovarian cancer. Anticancer Res..

[25] Du P., Wang Y., Chen L., Gan Y., Wu Q. (2016). High ERCC1 expression is associated with platinum-resistance, but not survival in patients with epithelial ovarian cancer. Oncol. Lett..

[26] Usanova S., Piée-Staffa A., Sied U., Thomale J., Schneider A., Kaina B., Köberle B. (2011). Cisplatin sensitivity of testis tumour cells is due to deficiency in interstrand-crosslink repair and low ercc1-xpf expression. J. Urol..

[27] Allingham-Hawkins D., Lea A., Levine S. (2010). ERCC1 expression analysis to guide therapy in non-small cell lung cancer. PLoS Curr..

[28] Rosell R., Lord R.V.N., Taron M., Reguart N. (2002). DNA repair and cisplatin resistance in non-small-cell lung cancer. Lung Cancer.

[29] Jiang J., Liang X., Zhou X., Huang R., Chu Z., Zhan Q. (2012). ERCC1 expression as a prognostic and predictive factor in patients with non-small cell lung cancer: A meta-analysis. Mol. Biol. Rep..

[30] Jun H.J., Ahn M.J., Kim H.S., Yi S.Y., Han J., Lee S.K., Ahn Y.C., Jeong H.S., Son Y.I., Baek J.H. (2008). ERCC1 expression as a predictive marker of squamous cell carcinoma of the head and neck treated with cisplatin-based concurrent chemoradiation. Br. J. Cancer.

[31] Weilbeer C., Jay D., Donnelly J.C., Gentile F., Karimi-Busheri F., Yang X., Mani R.S., Yu Y., Elmenoufy A.H., Barakat K.H. (2022). Modulation of ERCC1-XPF Heterodimerization Inhibition via Structural Modification of Small Molecule Inhibitor Side-Chains. Front. Oncol..

[32] Gentile F., Elmenoufy A.H., Ciniero G., Jay D., Karimi-Busheri F., Barakat K.H., Weinfeld M., West F.G., Tuszynski J.A. (2020). Computer-aided drug design of small molecule inhibitors of the ERCC1-XPF protein–protein interaction. Chem. Biol. Drug Des..

[33] Heyza J.R., Arora S., Zhang H., Conner K.L., Lei W., Floyd A.M., Deshmukh R.R., Sarver J., Trabbic C.J., Erhardt P. (2018). Targeting the DNA repair endonuclease ERCC1-XPF with green tea polyphenol epigallocatechin-3-gallate (EGCG) and its prodrug to enhance cisplatin efficacy in human cancer cells. Nutrients.

[34] Arora S., Heyza J., Zhang H., Kalman-Maltese V., Tillison K., Floyd A.M., Chalfin E.M., Bepler G., Patrick S.M. (2016). Identification of small molecule inhibitors of ERCC1-XPF that inhibit DNA repair and potentiate cisplatin efficacy in cancer cells. Oncotarget.

[35] Elmenoufy A.H., Gentile F., Jay D., Karimi-Busheri F., Yang X., Soueidan O.M., Weilbeer C., Mani R.S., Barakat K.H., Tuszynski J.A. (2019). Targeting DNA Repair in Tumor Cells via Inhibition of ERCC1-XPF. J. Med. Chem..

[36] Jordheim L.P., Barakat K.H., Heinrich-Balard L., Matera E.L., Cros-Perrial E., Bouledrak K., El Sabeh R., Perez-Pineiro R., Wishart D.S., Cohen R. (2013). Small molecule inhibitors of ercc1-xpf protein-protein interaction synergize alkylating agents in cancer cellss. Mol. Pharmacol..

[37] Chapman T.M., Gillen K.J., Wallace C., Lee M.T., Bakrania P., Khurana P., Coombs P.J., Stennett L., Fox S., Bureau E.A. (2015). Catechols and 3-hydroxypyridones as inhibitors of the DNA repair complex ERCC1-XPF. Bioorganic Med. Chem. Lett..

[38] Ghazy S., Oktay L., Durdagi S. (2024). A Novel Algorithm for the Virtual Screening of Extensive Small Molecule Libraries Against ERCC1/XPF Protein-Protein Interaction for the Identification of Therapeutic Resistance-Bypassing Small Anticancer Molecules. Turkish J. Biol..

[39] Manguinhas R., Serra P.A., Soares R.B., Rosell R., Gil N., Oliveira N.G., Guedes R.C. (2024). Unveiling Novel ERCC1–XPF Complex Inhibitors: Bridging the Gap from In Silico Exploration to Experimental Design. Int. J. Mol. Sci..

[40] Graf N., Ang W.H., Zhu G., Myint M., Lippard S.J. (2011). Role of Endonucleases XPF and XPG in Nucleotide Excision Repair of Platinated DNA and Cisplatin/Oxaliplatin Cytotoxicity. ChemBioChem.

[41] Walsh C.S., Ogawa S., Karahashi H., Scoles D.R., Pavelka J.C., Tran H., Miller C.W., Kawamata N., Ginther C., Dering J. (2008). ERCC5 is a novel biomarker of ovarian cancer prognosis. J. Clin. Oncol..

[42] Sung H., Ferlay J., Siegel R.L., Laversanne M., Soerjomataram I., Jemal A., Bray F. (2021). Global Cancer Statistics 2020: GLOBOCAN Estimates of Incidence and Mortality Worldwide for 36 Cancers in 185 Countries. CA Cancer J. Clin..

[43] Siegel R.L., Giaquinto A.N., Jemal A. (2024). Cancer statistics, 2024. CA Cancer J. Clin..

[44] Zappa C., Mousa S.A. (2016). Non-small cell lung cancer: Current treatment and future advances. Transl. Lung Cancer Res..

[45] Rose P.W., Prlic A., Altunkaya A., Bi C., Bradley A.R., Christie C.H., Di Costanzo L., Duarte J.M., Dutta S., Feng Z. (2017). The RCSB protein data bank: Integrative view of protein, gene and 3D structural information. Nucleic Acids Res..

[46] Barros A.C.D., Takeda A.A.S., Dreyer T.R., Velazquez-Campoy A., Kobe B., Fontes M.R.M. (2016). Structural and Calorimetric Studies Demonstrate that Xeroderma Pigmentosum Type G (XPG) Can Be Imported to the Nucleus by a Classical Nuclear Import Pathway via a Monopartite NLS Sequence. J. Mol. Biol..

[47] Tsutakawa S.E., Sarker A.H., Ng C., Arvai A.S., Shin D.S., Shih B., Jiang S., Thwin A.C., Tsai M.S., Willcox A. (2020). Human XPG nuclease structure, assembly, and activities with insights for neurodegeneration and cancer from pathogenic mutations. Proc. Natl. Acad. Sci. USA.

[48] González-Corrochano R., Ruiz F.M., Taylor N.M.I., Huecas S., Drakulic S., Spínola-Amilibia M., Fernández-Tornero C. (2020). The crystal structure of human XPG, the xeroderma pigmentosum group G endonuclease, provides insight into nucleotide excision DNA repair. Nucleic Acids Res..

[49] (2024). Molecular Operating Environment (MOE).

[50] Lucas S.D., Gonçalves L.M., Cardote T.A.F., Correia H.F., Moreira R., Guedes R.C. (2012). Structure based virtual screening for discovery of novel human neutrophil elastase inhibitors. Medchemcomm.

[51] Gaulton A., Hersey A., Nowotka M.L., Patricia Bento A., Chambers J., Mendez D., Mutowo P., Atkinson F., Bellis L.J., Cibrian-Uhalte E. (2017). The ChEMBL database in 2017. Nucleic Acids Res..

[52] Lagorce D., Sperandio O., Baell J.B., Miteva M.A., Villoutreix B.O. (2015). FAF-Drugs3: A web server for compound property calculation and chemical library design. Nucleic Acids Res..

[53] Verdonk M.L., Cole J.C., Hartshorn M.J., Murray C.W., Taylor R.D. (2003). Improved protein-ligand docking using GOLD. Proteins Struct. Funct. Genet..

[54] Korb O., Stützle T., Exner T.E. (2009). Empirical scoring functions for advanced Protein-Ligand docking with PLANTS. J. Chem. Inf. Model..

[55] Gareth J., Willett P., Glen R.C., Leach A.R., Taylor R. (1997). Development and validation of a genetic algorithm for flexible docking. J. Mol. Biol..

[56] Mooij W.T.M., Verdonk M.L. (2005). General and targeted statistical potentials for protein-ligand interactions. Proteins Struct. Funct. Genet..

[57] Marchand J.R., Pirard B., Ertl P., Sirockin F. (2021). CAVIAR: A method for automatic cavity detection, description and decomposition into subcavities. J. Comput. Aided. Mol. Des..

[58] ChemBridge. https://www.chembridge.com/.

[59] Bento A.P., Hersey A., Félix E., Landrum G., Gaulton A., Atkinson F., Bellis L.J., De Veij M., Leach A.R. (2020). An open source chemical structure curation pipeline using RDKit. J. Cheminform..

[60] van der Maaten L., Hinton G. (2008). Viualizing data using t-SNE. J. Mach. Learn. Res..

[61] Salentin S., Schreiber S., Haupt V.J., Adasme M.F., Schroeder M. (2015). PLIP: Fully automated protein-ligand interaction profiler. Nucleic Acids Res..

[62] Manguinhas R., Fernandes A.S., Costa J.G., Saraiva N., Camões S.P., Gil N., Rosell R., Castro M., Miranda J.P., Oliveira N.G. (2020). Impact of the APE1 redox function inhibitor E3330 in non-small cell lung cancer cells exposed to cisplatin: Increased cytotoxicity and impairment of cell migration and invasion. Antioxidants.

[63] Tumey L.N., Bom D., Huck B., Gleason E., Wang J., Silver D., Brunden K., Boozer S., Rundlett S., Sherf B. (2005). The identification and optimization of a N-hydroxy urea series of flap endonuclease 1 inhibitors. Bioorg. Med. Chem. Lett..

[64] Tumey L.N., Huck B., Gleason E., Wang J., Silver D., Brunden K., Boozer S., Rundlett S., Sherf B., Murphy S. (2004). The identification and optimization of 2,4-diketobutyric acids as flap endonuclease 1 inhibitors. Bioorg. Med. Chem. Lett..

[65] Ashrafi A., Akter Z., Modareszadeh P., Modareszadeh P., Berisha E., Alemi P.S., Chacon Castro M.C., Deese A.R., Zhang L. (2022). Current Landscape of Therapeutic Resistance in Lung Cancer and Promising Strategies to Overcome Resistance. Cancers.

