# Supplementary material for: Novel DNA Repair Inhibitors Targeting XPG to Enhance Cisplatin Therapy in Non-Small Cell Lung Cancer: Insights from In Silico and Cell-Based Studies
Source: Cancers (Basel). 2024 Sep 16;16(18):3174. doi: 10.3390/cancers16183174 (PMC11430689; doi:10.3390/cancers16183174)
Supplement: Supplementary file 1 [file cancers-16-03174-s001.zip › cancers-3161206-supplementary.pdf]

Supplementary Materials

CB9-G — 7636733	CB20-G — 51765644	CB22-G — 17938403
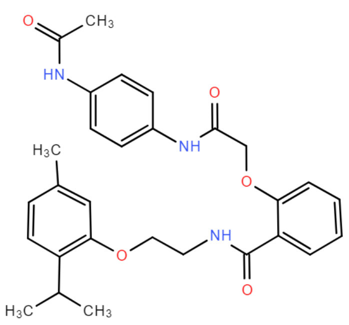	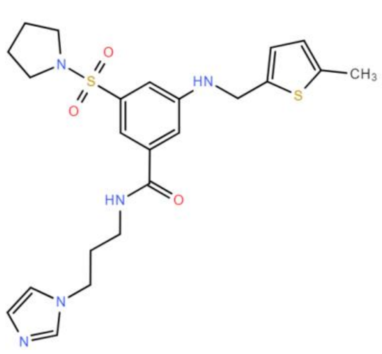	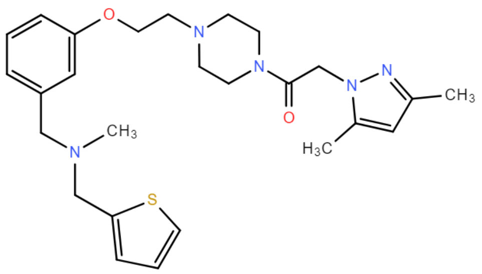
CB23-G — 87149755	CB41-G — 15324754	CB46-G — 5186642
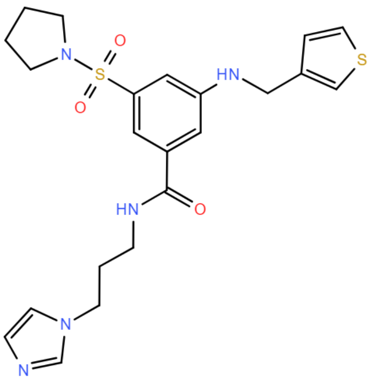	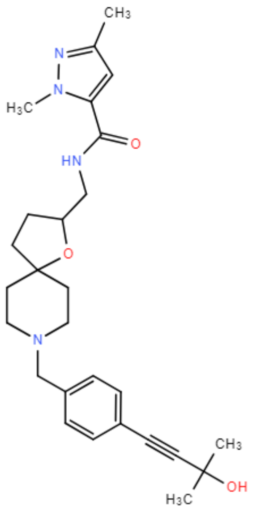	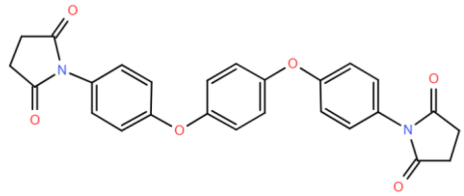
CB49-G — 73413968	CB60-G — 27740813	
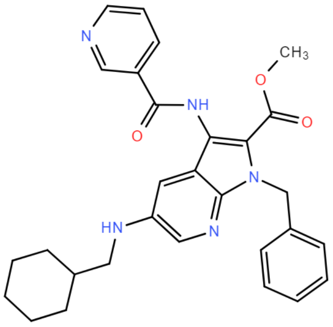	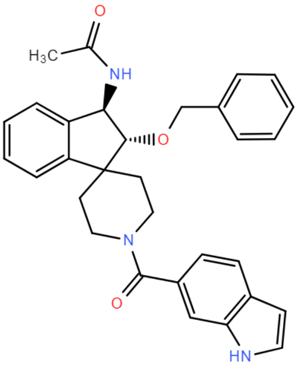	

Figure S1. List and chemical structures of the 8 compounds identified as best inhibitors from the ChemBridge database that could enhance cisplatin-induced cytotoxicity.

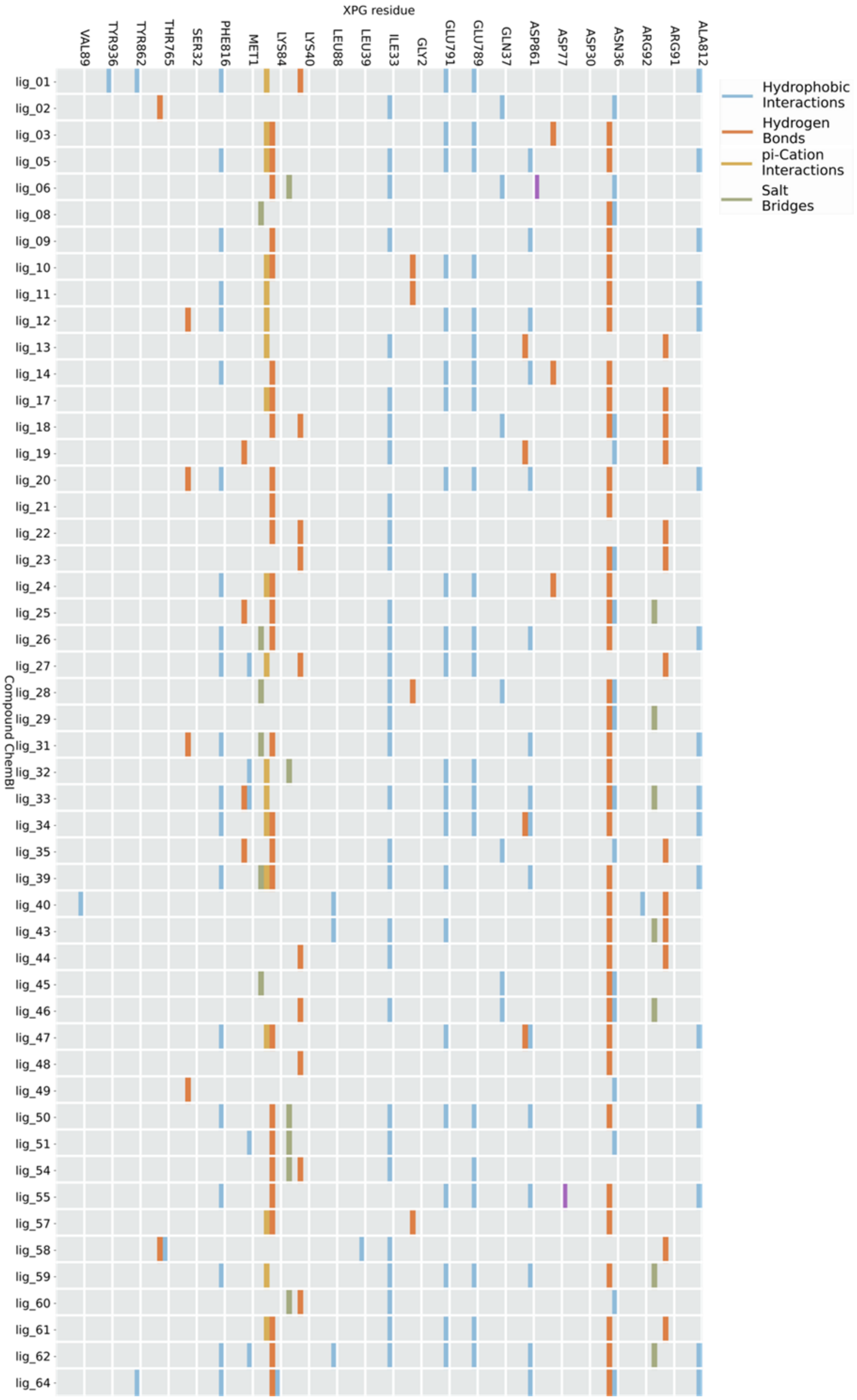

Figure S2. Representation of the types of interactions established between the XPG protein and the compounds retrieved from ChemBL with activity from 23 nM up to 30 μM (50 compounds). Each column represents the XPG residues, while the rows identify each of the 50 molecules. Our analysis considered various interactions, including H-bonds, hydrophobic interactions, π-stacking, water bridges, halogen bonds, and salt bridges. π-stacking interactions between these molecules and residues were not identified.

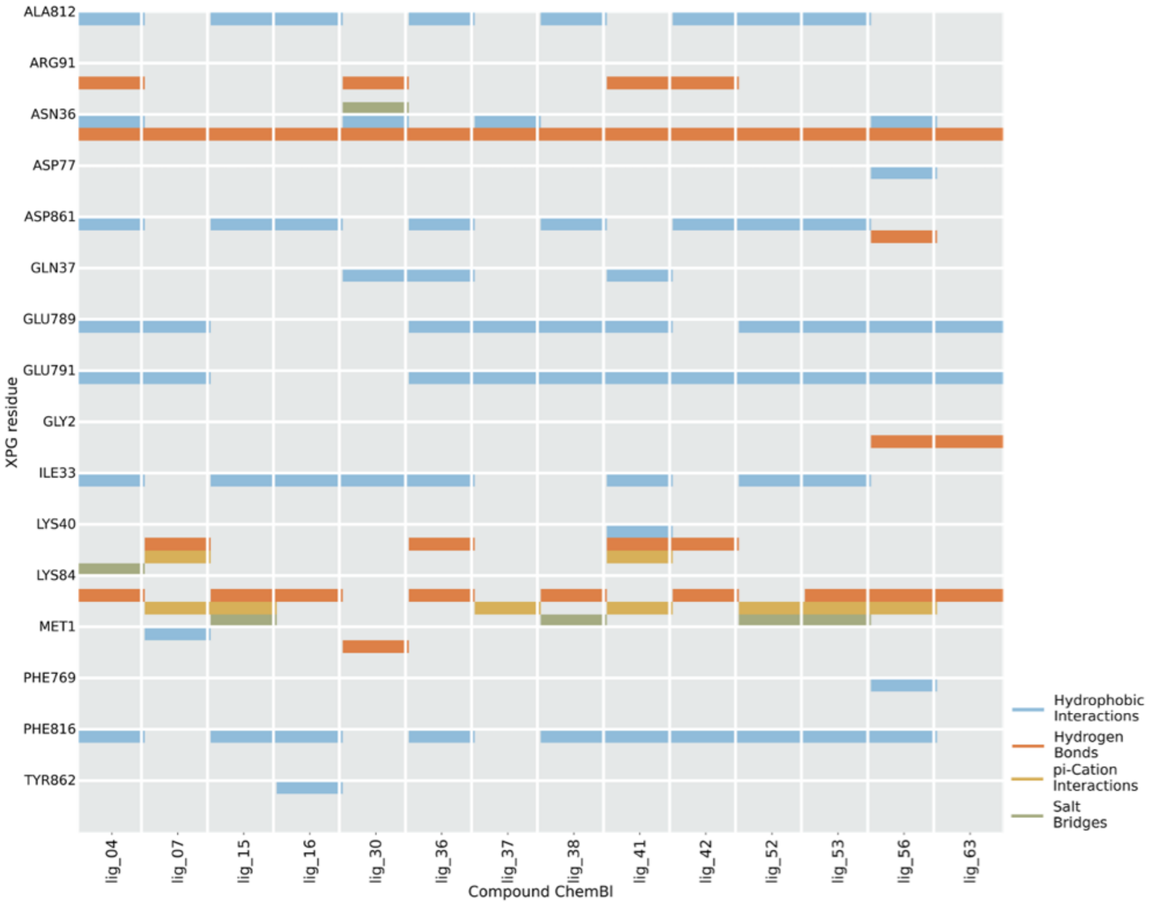

Figure S3. Representation of the types of interactions established between the XPG protein and the compounds from ChemBL with activity superior to 30 µM (14 compounds). Each row represents the XPG residues, while the columns identify each of the 50 molecules. Our analysis considered various interactions, including H-bonds, hydrophobic interactions, π-stacking, water bridges, halogen bonds, and salt bridges. π-stacking interactions between these molecules and residues were not identified.
